# A *Rhizobium leguminosarum* CHDL- (Cadherin-Like-) Lectin Participates in Assembly and Remodeling of the Biofilm Matrix

**DOI:** 10.3389/fmicb.2016.01608

**Published:** 2016-10-13

**Authors:** Nicolás F. Vozza, Patricia L. Abdian, Daniela M. Russo, Elías J. Mongiardini, Aníbal R. Lodeiro, Søren Molin, Angeles Zorreguieta

**Affiliations:** ^1^Fundación Instituto Leloir, IIBBA-CONICETBuenos Aires, Argentina; ^2^Instituto de Bioquímica y Biología Molecular, Departamento de Ciencias Biológicas, Facultad de Ciencias Exactas, Universidad Nacional de La Plata, Centro Científico Technológico COINCET La PlataLa Plata, Argentina; ^3^Department of Systems Biology, Technical University of DenmarkLyngby, Denmark; ^4^Departamento de Química Biológica, Facultad de Ciencias Exactas y Naturales, Universidad de Buenos AiresBuenos Aires, Argentina

**Keywords:** biofilms, extracellular matrix, lectins, exopolysaccharides, *Rhizobium*

## Abstract

In natural environments most bacteria live in multicellular structures called biofilms. These cell aggregates are enclosed in a self-produced polymeric extracellular matrix, which protects the cells, provides mechanical stability and mediates cellular cohesion and adhesion to surfaces. Although important advances were made in the identification of the genetic and extracellular factors required for biofilm formation, the mechanisms leading to biofilm matrix assembly, and the roles of extracellular proteins in these processes are still poorly understood. The symbiont *Rhizobium leguminosarum* requires the synthesis of the acidic exopolysaccharide and the PrsDE secretion system to develop a mature biofilm. PrsDE is responsible for the secretion of the Rap family of proteins that share one or two Ra/CHDL (cadherin-like-) domains. RapA2 is a calcium-dependent lectin with a cadherin-like β sheet structure that specifically recognizes the exopolysaccharide, either as a capsular polysaccharide (CPS) or in its released form [extracellular polysaccharide (EPS)]. In this study, using gain and loss of function approaches combined with phenotypic and microscopic studies we demonstrated that RapA lectins are involved in biofilm matrix development and cellular cohesion. While the absence of any RapA protein increased the compactness of bacterial aggregates, high levels of RapA1 expanded distances between cells and favored the production of a dense matrix network. Whereas endogenous RapA(s) are predominantly located at one bacterial pole, we found that under overproduction conditions, RapA1 surrounded the cell in a way that was reminiscent of the capsule. Accordingly, polysaccharide analyses showed that the RapA lectins promote CPS formation at the expense of lower EPS production. Besides, polysaccharide analysis suggests that RapA modulates the EPS size profile. Collectively, these results show that the interaction of RapA lectins with the polysaccharide is involved in rhizobial biofilm matrix assembly and remodeling.

## Introduction

It is now widely accepted that in most natural environments bacteria are found predominantly in biofilms (Davey and O’Toole, 2000; Danhorn and Fuqua, 2007). Biofilms are surface-associated multicellular communities, that initiate by attachment to a surface followed by production of a polymeric extracellular matrix. This extracellular matrix encloses bacteria, provides structural support and protection against desiccation and antimicrobials, and intensifies communication (Costerton et al., 1994; Branda et al., 2005; Flemming and Wingender, 2010). Although the genetics of the biofilm formation stages and the matrix composition of several bacterial species were extensively studied *in vitro*, how the biopolymers are assembled in a dynamic matrix is still poorly understood.

Rhizobia are remarkable bacteria because they can live either freely in soil or in the nitrogen fixing root nodules in symbiosis with legumes. However, most of the time rhizobia live in free living conditions on soil particles, in the rhizosphere or on plant roots. It was proposed that a biofilm life style is the optimal mode for rhizobia to persist and increase the chances of nodulation (Danhorn and Fuqua, 2007; Downie, 2010; Rinaudi et al., 2010). *Rhizobium* species can establish nitrogen-fixing symbiosis within root nodules with several legumes such as pea, lentil, bean, vetch, and clover. The observation that several surface and extracellular polysaccharides (EPSs) are produced by *Rhizobium leguminosarum* (Laus et al., 2006; Skorupska et al., 2006; Williams et al., 2008) suggests that different attachment mechanisms may be displayed according to the diverse niches and environmental conditions encountered by bacteria (Laus et al., 2006; Rodriguez-Navarro et al., 2007; Downie, 2010).

*Rhizobium leguminosarum* produces an acidic extracellular polysaccharide, formed by polymerization of octasaccharide repeating units containing glucose (Glc), glucuronic acid (GlcA), and galactose (Gal), in a 5:2:1 ratio with particular substitutions (Skorupska et al., 2006). Of note, inactivation of *pssA*, which is required for the first step in the synthesis of the repeating unit, also abolished the synthesis of the capsular polysaccharide (CPS) in *R. leguminosarum* bv *viciae* strains 3841 and A34 (Russo et al., 2006; Williams et al., 2008). This is not surprising because it has been reported that both polysaccharides share strong similarities in sugar composition and structure (Laus et al., 2004; Skorupska et al., 2006), but differ only in the relative proximity to the bacterial cell surface. The acidic polysaccharide (as EPS or CPS) is crucial for cell-cell interactions and biofilm formation (Fujishige et al., 2006; Russo et al., 2006; Williams et al., 2008).

The development of a mature and typical biofilm in *R. leguminosarum* bv. *viciae* strain A34 also requires a functional type I PrsDE secretion system (Russo et al., 2006). PrsDE is responsible for the secretion of Rap(s) (*Rhizobium* adhering-proteins), such as, the RapA paralogs, RapC, and the PlyA and PlyB glycanases, all of which share at least one Ra domain (Finnie et al., 1997; Ausmees et al., 2001; Russo et al., 2006; Krehenbrink and Downie, 2008). PlyA and PlyB modulate EPS chain lengths (and probably CPS chains) by cleaving the polymer molecules on the bacterial cell surface (Finnie et al., 1998; Zorreguieta et al., 2000). In consequence, mutants defective in the secretion of PlyB and PlyA form immature biofilms due to the production of unprocessed exopolysaccharide chains (Russo et al., 2006).

It was shown that RapA1 localizes at one pole of the bacterial surface and that addition of recombinant RapA1 to a planktonic culture of *R. leguminosarum* bv *trifolii* R200 promotes bacterial agglutination (Ausmees et al., 2001). Further investigations showed enhanced adhesion to clover roots when RapA1 was overproduced in *R. leguminosarum* R200 (Mongiardini et al., 2008). Moreover, in *R. leguminosarum* bv *viciae* strain 3841, high levels of Rap proteins, including RapA2, favor rhizobial attachment to pea roots and confer a competitive advantage for nodulation (Frederix et al., 2014). The RapA paralogs share approximately 90% similarity and are composed only of two Ra domains, which were predicted to display structural similarity to cadherin domains (Cao et al., 2005; Abdian et al., 2013). By biophysical methods we demonstrated that RapA2 indeed exhibits a calcium-dependent cadherin-like conformation. However, light scattering assays at different calcium concentrations and biochemical evidence showed that, unlike cadherins, RapA2 does not form oligomers. Instead, RapA2 specifically binds both the EPS and the CPS in a calcium-dependent manner (Abdian et al., 2013). In this work we aimed to give insight into the role of RapA lectins *in vivo*. Several observations indicate that the interaction of RapA proteins with the acidic polysaccharide influences adhesion to surfaces, cell-to-cell interaction, and matrix development. The evidences obtained herein, lead us to propose a role for the Rap family of proteins in capsule development and biofilm matrix remodeling.

## Materials and Methods

### Bacterial Strains and Growth Conditions

*Rhizobium leguminosarum* strains used in this work were *R. leguminosarum* bv *viciae* strain A34 (Downie et al., 1985), *R. leguminosarum* bv *viciae* strain 3841 (Johnston and Beringer, 1975; Young et al., 2006), *R. leguminosarum* bv *trifolii* strain R200 (Ausmees et al., 2001), and mutant derivatives from strain A34: *pssA*::Tn*5* (Km^r^; Russo et al., 2006) and *prsD*::Tn*5* (Km^r^; Finnie et al., 1997). Strains were grown in TY (Beringer, 1974) or Y-minimal medium (Sherwood, 1970) containing mannitol (0.2%, wt/vol) as the carbon source at 28°C with the appropriate antibiotics. Bacterial growth was monitored at 600 nm using an Ultrospec 1000 Pharmacia spectrophotometer (GE Healthcare, Piscataway, NJ, USA). Plasmids were mobilized into *Rhizobium* spp. by biparental mating using *Escherichia coli* S17 strain as donor (Simon et al., 1983). Working concentrations of antibiotics or dyes in culture media were as follows: streptomycin 400 μg/ml; kanamycin 50 μg/ml; tetracyclin 5 μg/ml; spectinomycin 200 μg/ml, gentamicin 10 μg/ml, and Congo red 25 μg/ml.

### Construction of the *rapA2* Mutant and the Complementing Plasmid

A deletion of *rapA2* (pRL100451) in strain 3841 was obtained by marker replacement, flanking the gene coding for gentamicin resistance by the upstream and downstream DNA regions of the *rapA2* gene. Two pairs of primers carrying sites for restriction endonucleases (rapA2-5up for TTAGAATTCAAACGGCTTCCAGCTCCTGG, rapA2-5up rev TATCTGCAGATTAGCGGGTCAATTTCGACCTCAG and rapA2-3down for AATGCATGCTTTGATCAGCAGTCATAGATCAGTG, rapA2-3down rev ATAAGCTTAAGCAGGCGACCAGGTCGAGC) were used to amplify a 525 bp DNA fragment, 22 bp upstream the *rapA2* initiation codon, or a 410 bp fragment including the 20 bp end of the *rapA2* gene and the complete sequence of a 69-amino acid putative protein. The gentamicin resistance gene was amplified with specific primers (genta for TATCTGCAGGACGCACACCGTGGAAAC and genta rev ACTGCATGCGCGGCGTTGTGACAATTTAC) from pBBR1MCS-5 (Kovach et al., 1995). The amplified products were sequentially cloned into pK19mobsacB (Schafer et al., 1994). First, the 525 bp upstream fragment was cloned into the EcoRI/PstI sites of pK19mobsacB, then the gentamicin resistance cassette was added to the construct at the PstI/SphI sites, and finally the 410 bp downstream fragment was cloned into the SphI/HindIII sites, resulting in pRapA2Mut. This plasmid was transferred from *E. coli* S17-1 to *R. leguminosarum* 3841 and simple cross-over events were selected on Y-minimal medium (to avoid *E. coli* growth) supplemented with streptomycin and kanamycin. Single colonies were picked from plates and grown on liquid cultures for 16 h. Finally, double cross-over events were selected on TY agar supplemented with 10% sucrose, streptomycin, and gentamicin. Kanamycin sensibility was checked on individual clones and gene replacement was confirmed by PCR.

The plasmid to complement the *rapA2* mutation was constructed on cosmid pLAFR3 (Staskawicz et al., 1987). A DNA fragment containing the *rapA2* gene and a non-coding sequence 500 bp upstream, was amplified by PCR using primers rapA2-5up for and rapA2-3down rev (see above). The 1680 bp product was cloned into the EcoRI/HindIII sites of pLAFR3 resulting in pLAF-*rapA2*.

### Biofilm Analyses

To observe the phenotypes of bacterial biofilms on semisolid media, cells were first grown on liquid TY medium with antibiotics at 28°C with shaking until late-exponential phase. The cultures were diluted with fresh medium to an OD at 600 nm of 0.05, and 25 μl were spotted on TY- 1.5% agar or Y- 1.5% agar plates supplemented with Congo red. Plates were incubated at 28°C for 3–4 days. Bacterial attachment to polystyrene was done as previously described (Russo et al., 2006). Bacteria were grown in TY medium (containing the appropriate antibiotics) for 2 days to an optical density at 600 nm of about 1.5. This culture was used as an inoculum at a 1:1000 dilution in Y-minimal medium and then pipetting 200 μl into the wells of polystyrene 96-well flat-bottom tissue culture plates (Corning Incorporated, Corning, NY, USA). The plates were cultured under static conditions for 5 days at 28°C. Unbound bacteria were removed by gently washing the wells three times with fresh growth medium, and attached bacteria were quantified by staining with 0.01% (wt/vol) Crystal violet (Acros Organics, Geel, Belgium), as described previously (O’Toole et al., 1999). Each strain was tested in three independent experiments with six replicate wells for each strain.

The biofilm developed on semisolid TY-media as a macrocolony was visualized by CSLM with a Carl Zeiss PASCAL LSM 5 using GFP detection settings and a C-Apochromat 63x/1.2 Water Objective. CSLM examination of the macrocolony structures was performed by placing an agar block containing the macrocolony in a glass slide with a single cavity (cavity-slide).

To analyze biofilm development, GFP-labeled bacteria carrying pHC60 (Cheng and Walker, 1998) or pHCrapAS (Mongiardini et al., 2008) from 48 h-TY medium cultures were diluted 1:1000 in Y-minimal media and incubated in chambered cover glass slides containing a borosilicate coverglass base (Lab-Tek, cat no. 155411; Nunc, Rochester, NY, USA) for 4 days at 28°C as previously described (Russo et al., 2006). CSLM was used to visualize the biofilm formation after 4 days using a Plan-Apochromat 100x/1.4 Oil Objective and exciting with argon laser (488 nm) and setting BP 505-530 filter for GFP detection. CLSM images were acquired using the Zeiss LSM Image Browser software 4.2.01 (Carl Zeiss MicroImaging GmbH).

Bacterial sedimentation assay was performed with 48 h- Y-minimal medium cultures of the wild type strains harboring either pHC60 or pHCrapAS that were left standing without shaking for 10 h.

### Scanning Electronic Microscopy (SEM)

Single macrocolony biofilms grown on TY-agar plates were carefully cut from the plates with a sterile scalpel, and placed in a sterile glass Petri dish, in which they were fixed and dehydrated with graded ethanol series according to Fischer et al. (2012). Afterward, agar blocks containing single macrocolonies were submitted to critical point drying. Samples were mounted on stubs and sputter coated with gold. SEM images were obtained with a ZEISS SUPRA 40 microscope.

### Preparation of Antibodies

The rabbit polyclonal antiserum to the RapA2 protein was obtained from purified protein (Abdian et al., 2013), and the antiserum to PlyB glycanase, was prepared from renatured inclusion bodies. The inclusion bodies were obtained by overexpression of a truncated form of PlyB (lacking the carboxi terminal Ra/CHDL domain; Ausmees et al., 2001) in *E. coli* BL21 (DE3)/pPlyBT. Total cell extracts, obtained by disruption in a French press were centrifuged at 3,000 × *g* for 5 min at 4°C to remove aggregates. The inclusion bodies were recovered from the 3,000 × *g* supernatant as described (Abdian et al., 2000) and used to immunize rabbits according to established protocols.

### Analysis of Extracellular Proteins and Immunodetection

Rhizobia were grown for 48 h at 28°C in TY media until late-exponential phase. Culture supernatant proteins were concentrated by precipitation with 10% trichloroacetic acid as described previously (Economou et al., 1990), the trichloroacetic acid was extracted by washing with acetone. Proteins were solubilized in loading buffer, separated by 10% SDS-PAGE and visualized by Coomassie blue staining. Surface-associated proteins were prepared from equivalent amounts of cells as follows: cells were precipitated by centrifugation, washed, and re-centrifuged; then resuspended in PBS, incubated at 60°C for 5 min and vortexed thoroughly for 30 s. Cells were removed by centrifugation and proteins in the supernatant were precipitated with 1 volume of acetone for 16 h at -20°C. After centrifugation, the protein pellet was solubilized in loading buffer. Western blot was carried out using standard techniques. Briefly, after SDS-PAGE, proteins were transferred to PVDF membranes (Millipore, Billerica MA, USA) blocked with 5% milk in TBS-0.05% Tween, and incubated sequentially with primary and secondary antibodies. Primary antibody against RapA1 from *R. leguminosarum* bv *trifolii* strain R200 (Ausmees et al., 2001), RapA2 (Abdian et al., 2013), or PlyB proteins from *R. leguminosarum* bv *viciae* 3841 were revealed by an anti-rabbit secondary antibody conjugated with alkaline phosphatase (Amersham Pharmacia – GE Healthcare, Piscataway, NJ, USA) in reaction buffer supplemented with 1% v/v BCIP and 1% v/v NBT (Promega, Madison WI, USA).

### Immunofluorescence (IF) and CLSM

IF of GFP-labeled *R. leguminosarum* strains with RapA-antiserum was performed as described previously (Ausmees et al., 2001) with minor variations. Briefly, bacteria were grown in TY medium for 24 h at 28°C. A 1 ml sample of the culture was centrifuged at 3,000 × *g* during 10 min, the cell pellets were washed with PBS and incubated for 30 min in blocking buffer (PBS containing BSA 0.5 mg/ml). Cells were then incubated with RapA-antiserum (1:250 in blocking buffer) for 1 h, at room temperature. After three washes with PBS, cells were incubated with anti-rabbit Cy3-conjugated secondary antibody (1:160 in blocking buffer; Jackson Immuno Research, West Grove, PA, USA) for 30 min. Cells were washed twice with PBS and an aliquot of the cell suspension was mounted on a slide and observed by CSLM using the Plan-Apochromat 100x/1.4 Oil Objective. Images were acquired using a Carl Zeiss PASCAL LSM 5 confocal microscope (Zeiss, Oberkochen, Germany) setting the filter for GFP (BP 505–530- pseudocoloured green) or Cy3 detection (LP 560- pseudocoloured red). The brightness or contrast were slightly adjusted (less than 10%), using the Zeiss LSM Image Browser software 4.2.01 (Carl Zeiss MicroImaging GmbH) to reduce background or saturation.

### Polysaccharide Preparation and Analyses

EPS and CPS were obtained from culture supernatants or cell pellets as described previously (Abdian et al., 2013), after growing *R. leguminosarum* strains in Y-minimal medium at 28°C for 48 h. Polysaccharides were quantified by determination of hexoses by the sulfuric acid-anthrone method (Loewus, 1952) and determination of hexuronic acids by the *meta*-hydroxydiphenyl method (Filisetti-Cozzi and Carpita, 1991). Exopolysaccharides isolated from culture supernatants were fractionated by gel filtration chromatography in a Superose 6 HR 10/30 column (Amersham Biosciences). Prior to separation EPS samples were boiled for 10 min to coagulate proteins, and centrifuged at 10,000 × *g* for 30 min. Samples of the supernatants containing 2 mg/ml of EPS were loaded onto the column, previously equilibrated in 0.1 M sodium phosphate buffer, pH 7.0, containing 0.1 M NaCl. Fractions (1 ml) were collected and assayed for carbohydrates according to Loewus (1952).

To analyze sugar composition, 200 μl aliquots of stock solutions of EPS (12 mg/ml), were submitted to acid hydrolysis with 2 N trifluoroacetic acid (TFA) at 110°C for 4 h (Albersheim et al., 1967). In some samples 2.5 mM GlcA was included as an internal standard. Residual acid was removed by repeated evaporation under nitrogen stream. The samples were finally resuspended in D_2_O. ^1^H-nuclear magnetic resonance (NMR) spectra of the hydrolysates and of Glc, Gal, and GlcA (2.5 mM each) were recorded on a Bruker Avance 600-MHz spectrometer at 298 K, using DSS (4,4-dimethyl-4-silapentane-1-sulfonic acid) as an external standard. The spectra were processed with Bruker Top Spin pack, NMRpipe suite and Matlab. Identification of monosaccharides was confirmed by comparison with data available at the the Biological Magnetic Resonance Data Bank^1^: Glc (BMRB ID bmse 000015), Gal (BMRB ID bmse 000013), and Glucuronolactone (BMRB ID bmse 001023).

## Results

### Extracellular RapA Affects Biofilm-Associated Phenotypes

**FIGURE 1:**
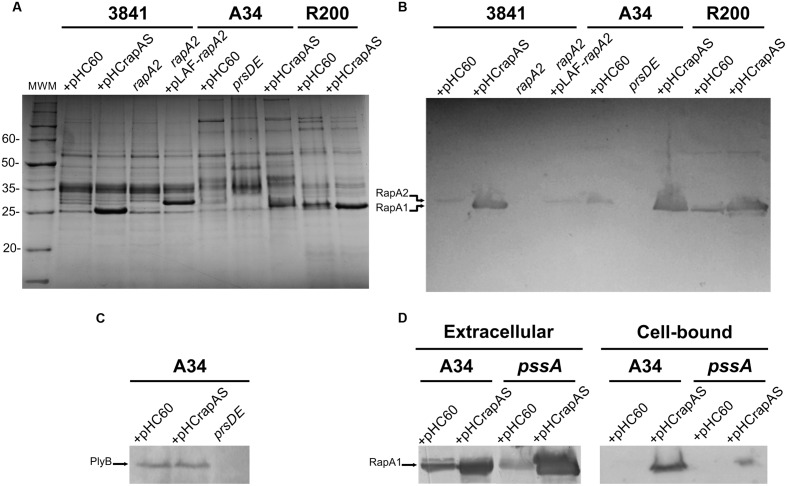
**Analysis of proteins secreted by the different genetic backgrounds.** **(A)** Extracellular proteins were precipitated from culture supernatants, separated by SDS-PAGE and visualized by Coomassie blue staining. The strains analyzed were: 3841, A34, and R200 wild type strains harboring the pHC60 empty vector or pHCrapAS, the *rapA2* mutant (in the 3841 background), the *rapA2* mutant complemented with the pLAF-*rapA2* plasmid, and the *prsD* secretion mutant. **(B)** Western blot using antibodies against the RapA1 protein. The arrows indicate bands corresponding to the RapA1 and RapA2 proteins. **(C)** PlyB secretion in the A34 background containing pHC60 or pHCrapAS was analyzed by Western blot using antibodies raised against the glycanase PlyB. The arrow indicates the PlyB protein band. **(D)** Western blot analysis of extracellular and surface-associated proteins from the *pssA* mutant harboring either pHC60 or pHCrapAS. Membranes were incubated with the anti-RapA1 antiserum. Representative blots are shown from three different protein preparations. Total protein was standardized using equal amounts of cultures with similar OD at 600 nm.

RapA proteins are composed of two Ra/CHDL domains, which confer specific lectin-binding activity toward the EPS and CPS of *R. leguminosarum* (Abdian et al., 2013). In addition, the Ply glycanases also harbor a Ra/CHDL domain in their carboxy-terminal end, which might participate in the binding of the enzyme to the polysaccharide for efficient processing. These observations, together with the fact that Rap proteins are co-secreted in a PrsDE-dependent manner lead to the hypothesis that RapA-EPS and RapA-CPS interactions probably influence polymer assembly or remodeling that ultimately impacts on biofilm matrix structure. Genomic analysis of the sequenced strain 3841 (Young et al., 2006) shows that this strain only encodes one putative functional RapA homolog (RapA2). Therefore, to find out the role of RapA lectins in matrix development an insertional mutant in the *rapA2* gene of 3841 was generated by double recombination. A gain-of-function approach was also carried out; thus a pHC60 (Cheng and Walker, 1998) derivative expressing *rapA1* under the *p_lac_* promoter (pHCrapAS; Mongiardini et al., 2008) was transferred by mating not only to the 3841 strain, but also to the A34 and R200 rhizobial strains. The level of RapA1 protein secretion was assessed in bacteria grown in TY-rich or Y-minimal media. Extracellular proteins were analyzed by SDS-PAGE and revealed by Coomassie blue staining. A strong band corresponding to RapA1 was observed in the extracellular protein fractions of 3841, A34, and R200 rhizobia harboring the pHCrapAS plasmid, grown in TY-medium (**Figure 1A**) or Y-minimal medium (not shown). By using the ImageJ software, we estimated that RapA1 secretion increased by about 10-fold in both media, compared with the level of RapA1 secretion in the control strains that contain the empty pHC60 vector. Western blot analysis confirmed the identity of RapA1 (**Figure 1B**). As expected, other RapA paralogs (such as RapA2), showing a slightly different molecular weight were detected in the extracellular media of A34 and R200 (**Figure 1B**).

PrsD and PrsE are the inner membrane ABC and the membrane fusion protein (MFP) components of the type I secretion system responsible for secretion of Rap proteins (Russo et al., 2006; Krehenbrink and Downie, 2008). To confirm that the prominent amount of RapA1 detected in the extracellular medium is mediated by an active secretion (and not by bacterial autolysis or other unspecific mechanism), we analyzed the extracellular proteins in the A34 isogenic *prsD* deficient strain carrying the pHCrapAS plasmid (Finnie et al., 1997). The *prsD*/pHCrapAS strain did not show any secretion of RapA1 (**Figures 1A,B**), indicating that bacterial lysis or leakage could not account for extracellular RapA1. Importantly, this observation indicates that even in overexpression conditions, RapA1 is exclusively secreted via PrsDE and not by other type I secretion systems harbored in *R. leguminosarum* genomes (Krehenbrink and Downie, 2008).

**FIGURE 2:**
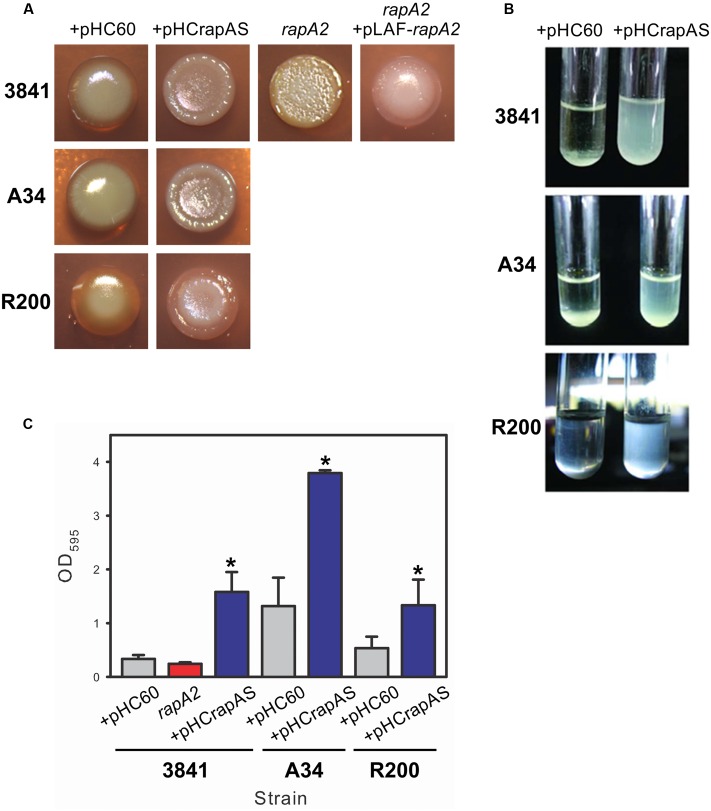
**Surface and biofilm phenotypes associated with RapA.** **(A)** Standarized bacterial suspensions of *Rhizobium leguminosarum* 3841, A34, R200, and isogenic derivative strains were spotted on TY-Congo red agar plates and cultured for 4 days at 28°C. **(B)** Bacterial sedimentation in Y-minimal medium was observed using standardized cultures of *R. leguminosarum* strains harboring either pHC60 or pHCrapAS that were previously cultivated until late-exponential phase and then left standing during 10 h. **(C)** Adhesion to polystyrene of bacteria grown in Y-minimal medium. At least three independent experiments were conducted with six replicate wells per experiment; the error bars indicate one standard deviation. Statistical analysis was performed using one-way analysis of variance (ANOVA) followed by Turkey *post hoc* test (Sigma plot version 11.0, Systat Software Inc, USA). ^∗^Significantly different from control (*P* < 0.05).

Bacteria growing on the surface of agar-solidified media are recognized as a particular type of biofilm that can show the ability to produce an extracellular matrix (Branda et al., 2005). When 25 μl of standardized liquid cultures of the 3841 strain and the isogenic *rapA2* mutant were spotted on TY-agar plates, different macroscopic phenotypes were clearly observed. While the wild type macrocolonies were brilliant, smooth, and dome shaped, the *rapA2* colonies were opaque, wrinkled, and flat. Besides, the *rapA2* gene cloned with its own promoter into the pLAFR3 vector restored the wild type colony appearance (**Figure 2A**). These observations suggest that the bacterial surface and/or the matrix characteristics are altered in the *rapA2* mutant. Interestingly, all the RapA1-overproducing strains formed wrinkled macrocolonies thickened at the borders, compared with the dome-shaped wild type colonies (**Figure 2A**).

**FIGURE 3:**
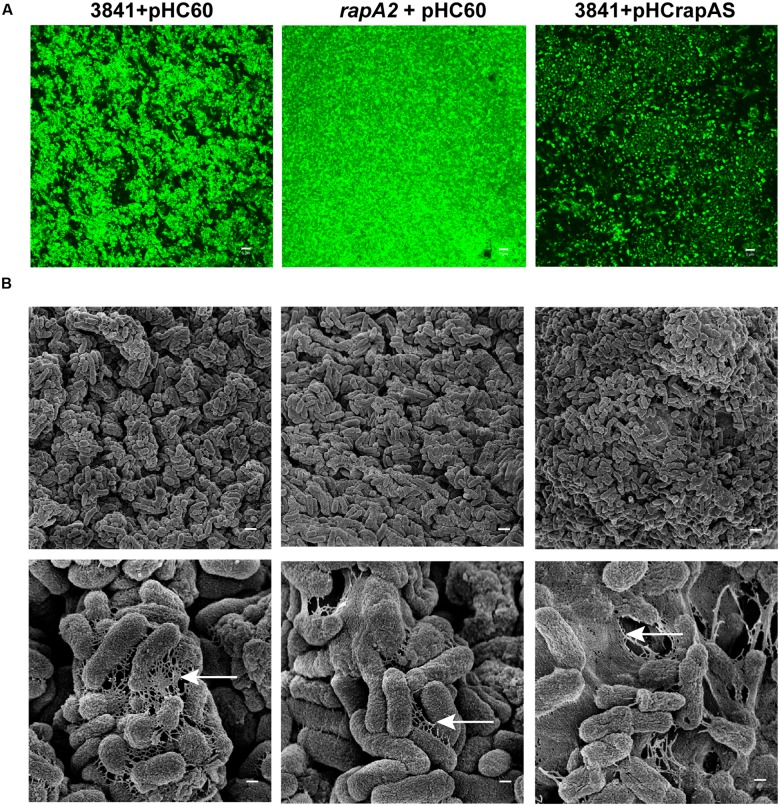
**Microscopic analysis of *Rhizobium* biofilms grown on semisolid medium.** 3841 derivative strains grown on TY-agar plates for 4 days were visualized by **(A)** CSLM and **(B)** Scanning Electronic Microscopy (SEM). **(A)** Biofilms of GFP-labeled 3841 pHC60, *rapA2* pHC60 and 3841 pHCrapAS strains are shown. Representative CSLM images from three independent experiments were obtained using a C-Apochromat 63x/1.2 Water Objective from a Carl Zeiss PASCAL LSM 5. White bar: 5 μm. **(B)** Cells and the matrix surrounding them in the biofilms formed by the wild type 3841 and derivative strains were observed by SEM in a ZEISS SUPRA 40 microscope. White bar: 1 μm, magnification at 10.000 X (upper panel). The disposition of the matrix among cells is indicated by arrows. White bar: 200 nm, magnification at 50.000 X (lower panel).

CSLM analysis of the TY-macrocolony biofilm showed that the *rapA2* mutant develops compact cellular aggregates with few void spaces in comparison with those of wild type 3841 (**Figure 3A**). In contrast, CLSM observation of the isogenic RapA1-overexpressing-biofilm indicated that increased RapA1 levels induce a low degree of cellular compactness (**Figure 3A**).

To further analyze the effect of the absence or the excess of RapA proteins in the interactions between cells and on the structure of the extracellular matrix, the biofilms grown on TY-agar were analyzed by SEM (**Figure 3B**). The 3841 strain developed well organized microcolonies, in which bacteria were interconnected by a network of fibrous extracellular matrix. In contrast, cells of the *rapA2* mutant strain formed compact microcolonies with scarce matrix around bacteria. Remarkably, the biofilm of the isogenic RapA1-overproducing strain was more disorganized with bacteria immersed in a dense mesh of extracellular material.

A distinct phenotype was also observed in liquid cultures of RapA1-overproducing strains grown in Y-minimal medium. After 10 h without shaking, most of these bacteria were still in suspension, while wild type cells settled in the bottom of the tube (**Figure 2B**). Overexpression of *rapA1* in the PrsDE-defective mutant, in contrast, did not produce any distinguishable phenotype in semisolid or liquid media (not shown), indicating that changes in these phenotypes are directly associated with RapA1 secretion.

Evaluation of bacterial attachment to an abiotic surface such as polystyrene provides a way to examine both the affinity of the cell surface for a hydrophobic surface and the ability of bacteria to interact with each other. The 3841-derivative *rapA2* mutant showed a slight and reproducible reduction in the attachment to polystyrene in comparison with the parental strain, although the difference was not statistically significant. To note, the 3841 strain *per se* showed a particular low ability to attach to polystyrene. However, high levels of RapA1 secretion in 3841 pHCrapAS resulted in threefold enhanced bacterial attachment to polystyrene in comparison with 3841 pHC60 (**Figure 2C**). Similarly, enhanced RapA1 secretion in the A34 and R200 wild type strains markedly increased bacterial attachment to the microtiter plate by about threefold to fourfold (**Figure 2C**).

**FIGURE 4:**
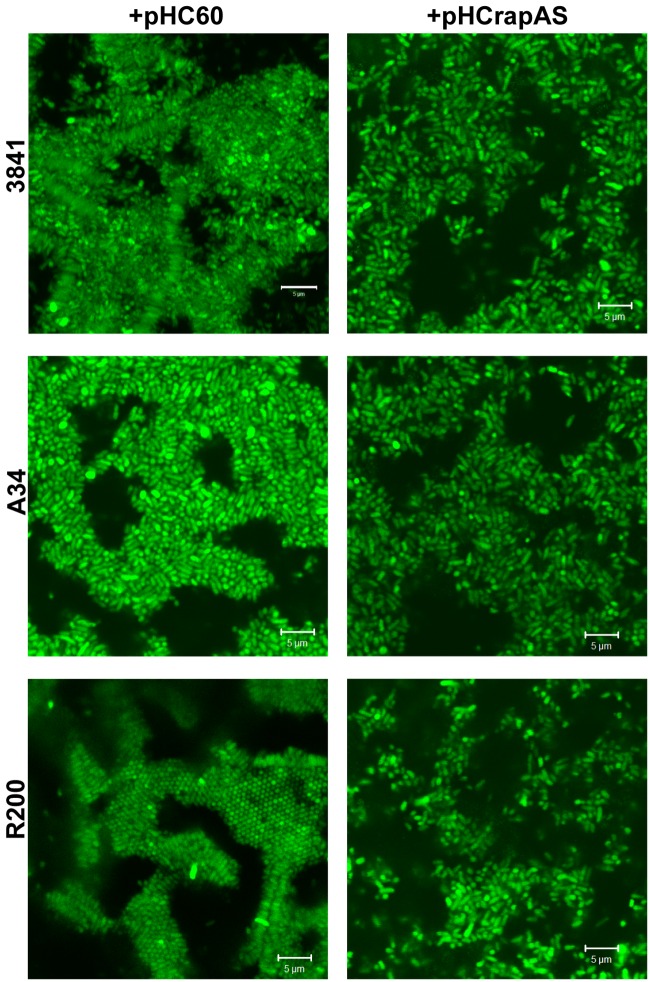
**RapA1 overproduction expands distances between bacterial cells.** CLSM images of 3841, A34, and R200 strains harboring pHC60 or pHCrapAS grown in chambered cover slides for 4 days in Y-minimal medium. Representative CSLM images of at least three different experiments observed using a Plan-Apochromat 100x/1.4 Oil Objective from a Carl Zeiss PASCAL LSM 5 are shown. White bar: 5 μm.

We have previously shown that GFP-labeled-rhizobia grown in liquid Y-minimal medium in chambered cover slides develop, after 2 days, microcolonies showing intimate lateral interactions, and after 4 days a typical biofilm structure (Russo et al., 2006). Under identical culture conditions, we did not detect altered cell-cell interactions or microcolony structures in the *rapA2* mutant compared with the 3841 isogenic wild type strain (data not shown). However, cellular interactions in RapA1-overexpressing rhizobia were altered; in fact, increased distances between bacterial cells were consistently observed in these strains (**Figure 4**).

Since PrsDE is responsible for secretion of several proteins, in addition to RapA paralogs, the possibility exists that high levels of RapA1 secretion may affect secretion of other Rap(s). For example, a reduction in the secretion of the CPS/EPS-glycanases (PlyA or PlyB) would have an effect in CPS/EPS length distribution, which in turn would alter some biofilm-associated phenotypes. However, except for RapA1 levels, no other differences in extracellular protein patterns were observed between RapA1-overexpressing rhizobia and the wild type strains (**Figure 1A**). Besides, Western blot analysis showed that PlyB secretion was not altered in the RapA1-overexpressing cells (**Figure 1C**). Therefore, it seems that the distinct phenotypes in the RapA1-overproducing strains are not due to a defect in the secretion of other PrsDE-dependent proteins.

Taken together, all these results show that the RapA lectins influence the characteristics of the extracellular matrix network and in consequence, the development of a robust biofilm.

### RapA Localization and Phenotypic Dependence on CPS/EPS Synthesis

**FIGURE 5:**
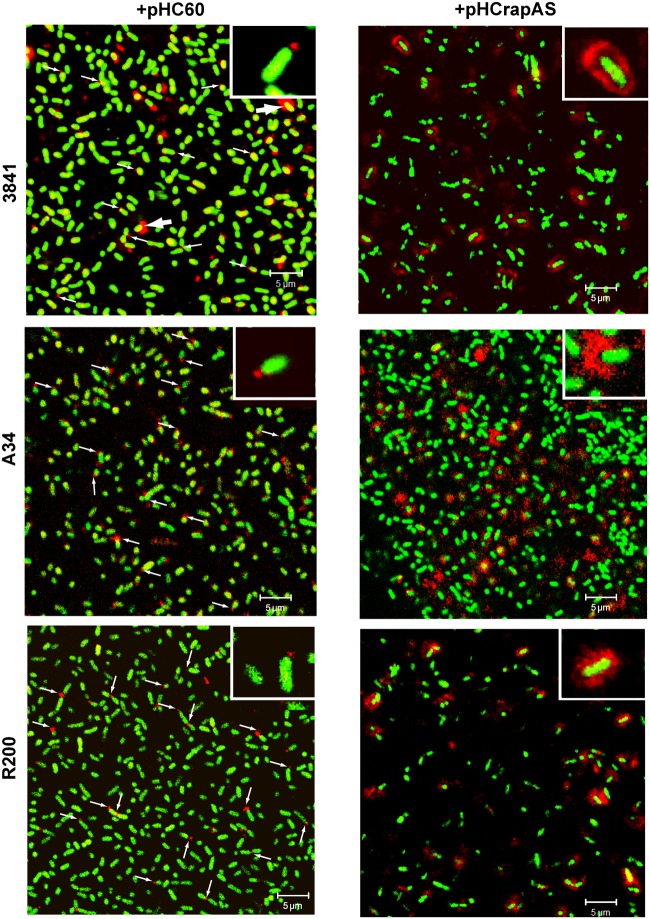
**RapA localization under endogenous and overexpressing conditions.** Immunofluorescence (IF) images acquired by CLSM of GFP-labeled strains (green) harboring pHC60 or pHCrapAS using anti-RapA2 antiserum and a Cy3-labeled secondary antibody (red) are shown. Thin arrows indicate polar localization of RapA. Thick arrows indicate capsule-like localization of RapA in the 3841 wild type strain. Details of polar (left) and capsule-like localization (right) are shown in the insets. Representative unbiased CSLM images of four different experiments using a Plan-Apochromat 100x/1.4 Oil Objective were acquired in a Carl Zeiss PASCAL LSM 5. White bar: 5 μm.

As it was previously reported for the R200 strain (Ausmees et al., 2001), IF and CLSM analysis showed that under conditions of endogenous expression numerous bacterial cells in the 3841 and A34 genetic backgrounds showed a RapA signal at one cell pole (**Figure 5**). Interestingly, some bacteria (typically in the 3841 strain, **Figure 5**) showed a fluorescent signal surrounding part or the entire cell, in a way that was reminiscent of the capsule. Furthermore, when RapA1 was overexpressed, a high proportion of bacteria in all the 3841, A34, and R200 genetic backgrounds showed this pattern; i.e., a strong red fluorescent signal surrounding the bacterial cell was observed. This effect can be easily explained by the lectin activity of RapA proteins toward the CPS (Abdian et al., 2013).

**FIGURE 6:**
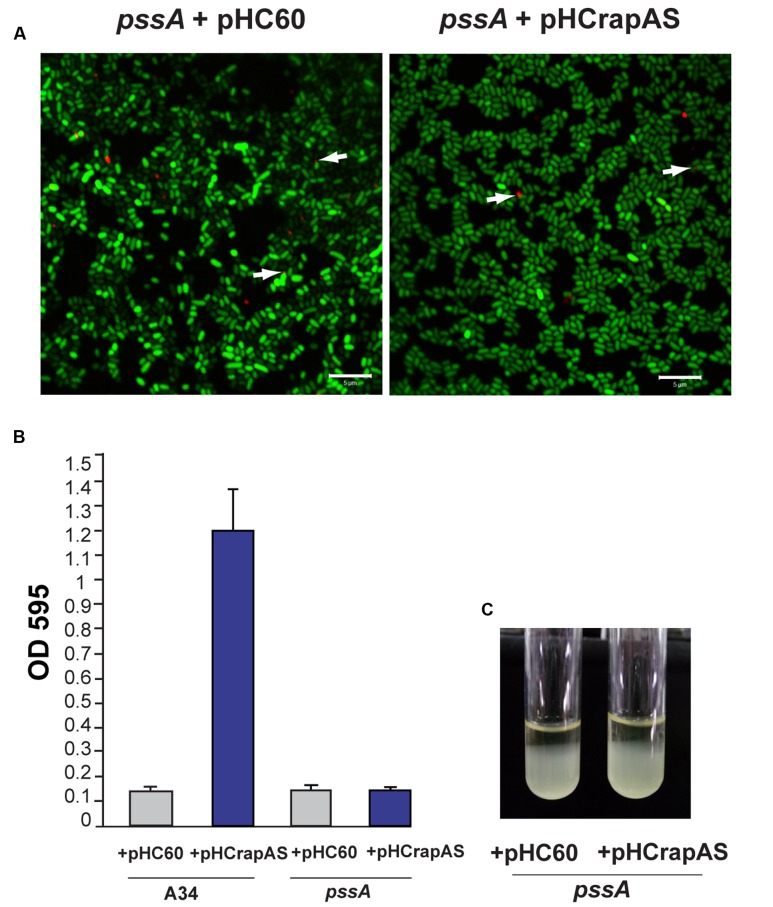
**RapA-associated phenotypes are dependent on CPS (capsular polysaccharide)/EPS (extracellular polysaccharide) synthesis.** **(A)** IF images of GFP-labeled *pssA* strains (green) harboring pHC60 or pHCrapAS using anti-RapA2 antiserum and Cy3-labeled secondary antibody (red) were acquired using a Plan-Apochromat 100x/1.4 Oil Objective from a CLSM. Arrows indicate polar localization of RapA. White bar: 5 μm. **(B)** Adhesion to polystyrene of bacteria grown in Y-minimal medium. At least three independent experiments were conducted with six replicate wells per experiment, the error bars indicate one standard deviation. **(C)** Bacterial sedimentation in Y-minimal medium cultures. *R. leguminosarum* strains harboring either pHC60 or pHCrapAS were left standing for 10 h.

To confirm that the phenotypes associated to RapA1-overproduction were dependent on CPS/EPS synthesis, the CPS/EPS-defective *pssA* mutant (Russo et al., 2006) and the isogenic A34 parental strain, harboring the vector pHC60, or the pHCrapAS were phenotypically analyzed. We first checked that RapA1 secretion was not impaired in the CPS/EPS-defective mutant. Indeed, Western blot analysis showed a strong RapA1 signal in the extracellular medium of the *pssA* mutant (**Figure 1D**). Furthermore, when RapA1 was overexpressed, a prominent RapA1 signal was consistently observed in the extracellular protein fraction of the *pssA* mutant strain (**Figure 1D**), strongly suggesting that these cells are unable to hold RapA1 on the cell surface. In line with these observations, a very small amount of cell surface-associated RapA1 was observed in *pssA* pHC60 or *pssA* pHCrapAS, compared with A34 pHC60 and A34 pHCrapAS, respectively (**Figure 1D**). Besides, IF and CSLM analysis of *pssA* pHC60 cells showed a strong reduction in the number of bacteria with polar RapA1 signal and the capsule-like pattern was absent in *pssA* pHCrapAS (**Figure 6A**). Accordingly, RapA1 overproduction neither changed the ability to attach to polystyrene (**Figure 6B**), the sedimentation (**Figure 6C**), nor the colony phenotypes (not shown), in the *pssA* mutant.

In conclusion, these observations indicate that the cell surface and biofilm matrix phenotypes attributed to RapA1 overproduction are strictly dependent on CPS/EPS production, supporting the lectin function of RapA toward the CPS/EPS *in vivo*.

### RapA Influences CPS/EPS Balance and EPS Size Distribution

**FIGURE 7:**
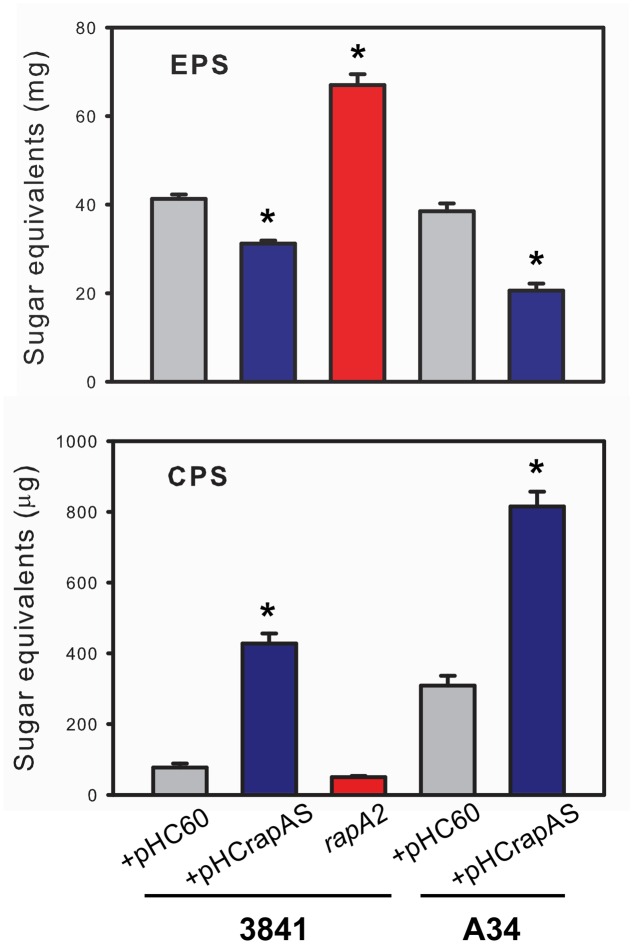
**Quantification of EPS and CPS.** The EPS and CPS produced by the 3841 and A34 strains and their derivatives were prepared from liquid cultures in Y-minimal medium as described in the text. Quantification was performed by determination of hexoses by the sulfuric acid-anthrone method. The results are representative of two independent experiments that were measured in triplicate each time, with similar results. Data was normalized to mg of cells (fresh weight) and evaluated by ANOVA followed by Turkey *post hoc* test. (Sigma plot version 11.0, Systat Software Inc., USA). ^∗^Significantly different from control (*P* < 0.05).

**FIGURE 8:**
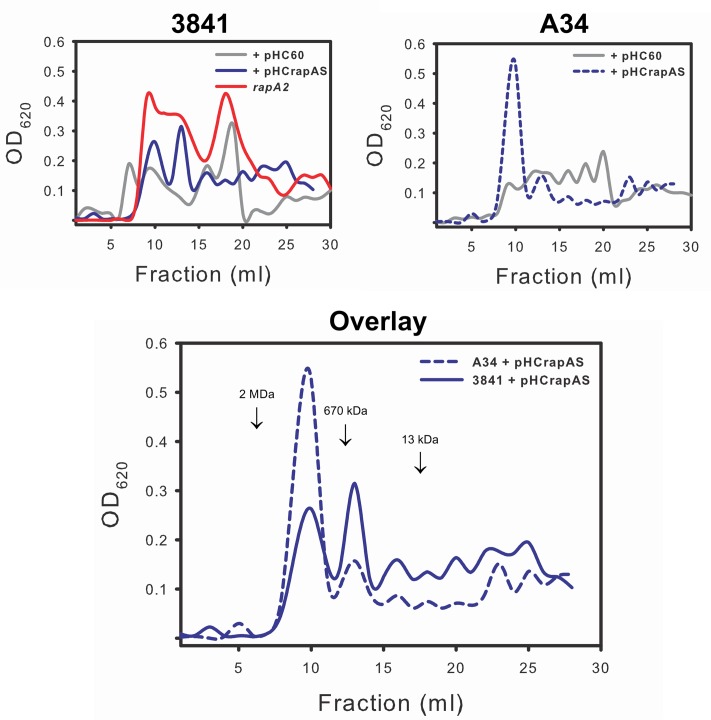
**Analysis of the EPS profiles by gel filtration chromatography.** EPS produced by strains 3841 and A34 harboring pHC60 or pHCrapAS and the *rapA2* mutant were fractionated on a Superose 6 column as described in the text. The elution volume of molecular mass markers is indicated by arrows. The same observation was made in two different and independent experiments.

The above observations are consistent with the notion that the interaction of RapA proteins with the acidic polysaccharide on the cell surface influences biofilm-related phenotypes. Although these phenotypes could be ascribed to a direct effect of such interaction, other non-exclusive possibility is that this interaction modifies some characteristics of the polysaccharide in its released form or as capsule. To analyze this possibility, the EPS and the CPS were obtained from the extracellular medium and the cell surface, respectively, of A34 and 3841 strains, the *rapA2* mutant and the isogenic strains that overproduce RapA1. Briefly, bacteria were grown in Y-mannitol-medium to favor polysaccharide production; polymers were prepared as described under “Materials and Methods” section and quantified by determination of hexoses and hexuronic acid. Interestingly, the RapA-overproducing strains produced a threefold to fivefold increase in the levels of CPS, while the amount of EPS showed a 30–50% reduction compared with the isogenic wild type strains. Conversely, the *rapA2* mutant showed the opposite balance in CPS/EPS production (**Figure 7**). The higher CPS level in the RapA1 overproducing strains could account for the RapA capsule-like distribution around the cell observed by IF-CLSM (**Figure 5**). We also analyzed the effect of the absence or the overproduction of RapA1 in the polysaccharide size distribution by size exclusion chromatography (SEC), using a Superose 6 column. To note, the relative amount of EPS recovered from the culture supernatants of all the strains was from 25- to 500-fold higher than the amount of CPS extracted from cells (**Figure 7**). Therefore, we concentrated our efforts in the analysis by SEC of the EPS samples. The 3841 pHC60 and A34 pHC60 genetic backgrounds produced heterogeneous and different EPS profiles (**Figure 8**). Although the *rapA2* mutant and the RapA1-overproducing strains showed different EPS patterns in comparison with the corresponding isogenic wild type strains, we did not observe a RapA-dependent consistent variation (i.e., a decrease or an increase) in the molecular size distribution of the polymer species (**Figure 8**). However, when the EPS profiles of 3841 pHCrapAS and A34 pHCrapAS were superimposed, it was clear that a particular EPS pattern was produced in both cases (**Figure 8**).

**FIGURE 9:**
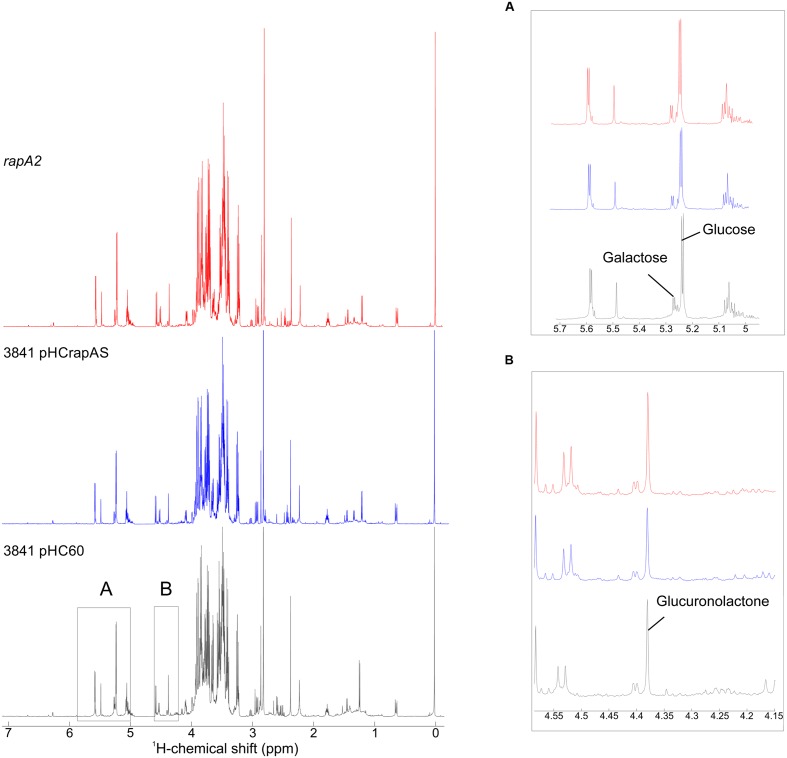
**^1^H NMR spectra of hydrolyzed samples of EPS.** Purified EPS from strains 3841 harboring pHC60 or pHCrapAS and the *rapA2* mutant were hydrolyzed under acidic conditions and analyzed by ^1^H NMR spectroscopy. Insets show expanded areas of the spectra. **(A)** The identification of Glc, Gal, and GlcA was performed using standard commercial sugars. The peaks at δ 5.241 and 5.234 correspond to Glc, while the signals at δ 5.273 and 5.266 are characteristic of Gal. Determination of the ratio Glc:Gal was done by integration of peak areas, resulting in 5.02 (3841 pHC60), 4.98 (3841 pHCrapAS), and 4.95 (*rapA2*). **(B)** The signal at δ 4.38 corresponds to the lactone form of GlcA obtained under the assay hydrolysis conditions.

The sugar composition of the EPS produced by 3841 harboring empty pHC60 and its isogenic strains, 3841 pHCrapAS and the *rapA2* mutant, were analyzed by NMR spectroscopy after acid hydrolysis of the samples. Similar spectral profiles were obtained for all the EPS hydrolyzed samples, irrespective of the levels of RapA expression (**Figure 9**). We also found that the ratios of Glc and Gal remained similar to that expected for the acidic EPS (5:1) in all cases (**Figure 9A**). It should be noted that the precise quantification of GlcA residues is difficult due to the stability of the β-(1,4) glycosidic bond between two GlcA (De Ruiter et al., 1992; Heyraud et al., 1993) and the transformation of GlcA into glucuronolactone under our hydrolysis conditions (Wang et al., 2010). However, GlcA was included as an internal standard in some samples, which let us identify easily the peaks corresponding to the lactone derivative of GlcA in the samples (**Figure 9B**). Identification of Glc and Gal was done by comparison with the spectra obtained for monosaccharide standards and also with those at the Biological Magnetic Resonance Data Bank.

Taken together, all these results suggest that RapA proteins do not affect EPS composition, but instead influence the balance CPS/EPS and also EPS processing in the extracellular milieu.

## Discussion

Although significant advances have been made in the identification of the biofilm matrix components and the genetic requirements to develop mature biofilms in several pathogenic and environmental relevant bacteria, the mechanisms involved in matrix assembly and remodeling are little understood (Flemming and Wingender, 2010). In this work, we describe the role of a polysaccharide–binding lectin in biofilm matrix assembly. Rap proteins were originally identified by their ability to attach to the rhizobial surface by screening of a M13 phage display library of the *R. leguminosarum* bv. *trifolii* R200 genome. With this approach, four Rap(s) were identified: the RapA1 and RapA2 paralogs, RapB and RapC (Ausmees et al., 2001). Later, it was reported that the A34 and 3841 strains of *R. leguminosarum* bv. *viciae* secrete a variable number of Rap(s), along with the PlyA and PlyB EPS-glycanases by the PrsDE type I protein secretion system (Russo et al., 2006; Krehenbrink and Downie, 2008). We have previously proposed to consider the Ply glycanases as Rap(s) since they also harbor a Ra domain (Abdian et al., 2013); but in this case, it was clearly demonstrated that they participate in polysaccharide processing on the cell surface once the polymer has been synthesized and exported (Zorreguieta et al., 2000). Therefore, aside from these glycanases, RapA proteins are so far the only Rap that has been characterized in more detail; in fact, it was demonstrated that RapA proteins are calcium-binding lectins composed of two CHDL (or Ra) domains that specifically interact with the EPS and CPS *in vitro* (Abdian et al., 2013). Accordingly, here we present several evidences showing that such interaction also occurs *in vivo*. Indeed, our results indicate that RapA paralogs influence the rhizobial adhesive properties, cell-cell interactions, and the biofilm matrix strength in an EPS/CPS-dependent fashion.

Several surface-related phenotypes were altered in a rhizobial strain that completely lacks a RapA protein and in three different *Rhizobium* genetic backgrounds that overproduce a RapA paralog. It was evident that the absence of any RapA protein in the 3841-derivative mutant changed the macroscopic phenotype of the biofilm grown on semisolid medium. Furthermore, microscopic analyses showed that mutant cells within these biofilms are tightly packed and scarce fibrous material was observed around the cells in comparison with the parental strain. On the contrary, high levels of extracellular RapA increased distances between bacterial cells, which were observed to be immersed in a dense net of extracellular matrix. IF analysis showed that, under conditions of endogenous expression, RapA proteins are predominantly detected at one pole, although some bacteria showed a more diffuse pattern around the cells. When RapA1 was overproduced the fluorescent capsule-like pattern was clearly observed in numerous bacterial cells. In line with these observations, polysaccharide quantification demonstrated a threefold to fivefold increase in the amount of CPS associated to RapA1-overexpressing cells. The low sedimentation rate of the RapA1-overexpressing bacterial cells in comparison with the wild type parental strains clearly supports the notion that the cell surface features are altered in these bacteria. However, in light of the overall picture, the interpretation of this phenotype is not easy. One possible explanation is that the low sedimentation rate is related to a reduction in the cellular aggregate compactness in the RapA-overexpressing cultures due to the exacerbated capsule production and the expanded cell-cell distances.

The positive effect of RapA on the CPS/EPS ratio was an intriguing observation. As stated earlier, the main difference between both polysaccharides is their closeness to the bacterial cell surface; while the EPS is completely liberated to the extracellular milieu, the CPS is retained on the cell surface by an unknown mechanism. In addition, it was reported that the EPS and the CPS differ in the degree of non-glycosidic substituents; the amounts of acetate, pyruvate, and 3-hydroxybutyrate were higher in the CPS than in the EPS (Hollingsworth et al., 1984). This and other unknown differences (such as the chain sizes) might have an impact on the physicochemical properties of polymer molecules that emerge from the cell surface that ultimately will define their destination. Therefore, the RapA lectins could favor (directly or indirectly) the formation of a complex network of polysaccharide around the cell. It was surprising that high levels of RapA somehow homogenized the EPS profiles in two different *Rhizobium* strains. An attractive hypothesis is that the interaction of emerging polysaccharide chains with RapA lectins influences the activity of PlyA and PlyB on the polysaccharide. In this scenario, high levels of RapA would favor the production of a particular range of polymer sizes.

It was shown that the absence of the exposed moiety of the LPS strongly affects cell-cell cohesion and the formation of a compact biofilm in *R. leguminosarum* (Russo et al., 2015). Indeed, the core-O antigen moiety of the LPS was crucial for lateral cell-cell interactions. Therefore, it would be interesting to find out whether the role of RapA-CPS/EPS interaction on cellular cohesion and biofilm matrix development is influenced by the exposed portion of the LPS.

Type I secretion systems in other species usually secrete one or few related substrates (Omori and Idei, 2003). PrsDE is a unique type I secretion system because it is responsible for secretion of a considerable number of protein substrates: at least nine proteins in strain A34 (Finnie et al., 1997; Russo et al., 2006) and up to 11 in strain 3841 (Krehenbrink and Downie, 2008), most of them of unknown function. Typically, proteins secreted by type I secretion systems are genetically associated with the secretion locus (Omori and Idei, 2003). This is certainly the case of *plyA* and *rapA1*, which are positioned very close to the chromosomal *prsD prsE* secretion locus, but not of *plyB*, which is more than 600 kb upstream *prsD prsE* (in 3841), or *rapA2*, which is not encoded by the bacterial chromosome but by the pRL10 plasmid (pSym) of *R. leguminosarum* 3841. Analysis of the 3841 genome indicates that the other *rap* genes are not close to the *prsDE* secretion locus or to a *pss* polysaccharide synthesis cluster. However, this observation does not rule out their functional relationship with the EPS/CPS. Thus, the possibility of other PrsDE substrates, in addition to Ply and RapA proteins, sharing a role in biofilm matrix development sounds plausible. We are currently performing biochemical studies with other Rap(s) to get a comprehensive picture of the functions of this family of proteins on the EPS/CPS structure and biofilm development.

Few reports describe the presence of proteins with or without enzymatic activity associated with the biofilm matrix. It was proposed that the presence of enzymes that degrade extracellular polymeric substances makes the matrix an external digestive system that breaks down biopolymers to small products that can be utilized as carbon and energy sources. In addition, some matrix-associated enzymes might be involved in the degradation of the biofilm matrix to promote bacterial detachment from biofilms (Flemming and Wingender, 2010). Some reports described the role of non-enzymatic proteins associated to the matrix, such as lectins. The secreted protein CdrA was shown to bind directly to the Psl polysaccharide in *Pseudomonas aeruginosa* biofilms, leading to the hypothesis that extracellular CdrA cross-links Psl molecules and thus strengthens the matrix, whereas cell-associated CdrA anchors the cells to Psl in the matrix (Borlee et al., 2010). Interestingly, multiresolution imaging of living *Vibrio cholerae* biofilms showed complementary roles of several matrix-associated proteins and the exopolysaccharide (VPS); the RbmA protein was involved in cell-cell adhesion and cluster formation and the VPS was required for accumulation on the cell surface of RbmA and other matrix associated proteins (Berk et al., 2012). Recently, a calcium-binding protein (CabA) was shown to contribute to biofilm maturation of the food-borne pathogen *Vibrio vulnificus*. It was shown that CabA is a matrix-associated protein that can assemble a functional matrix only when exopolysaccharides coexist (Park et al., 2015). We found that the interaction between the rhizobial RapA lectins and the CPS/EPS influences the biofilm matrix strength. Since the Rap proteins share one or two polysaccharide-binding domains, our working hypothesis is that assembly or remodeling of a biofilm matrix could be modulated by several of these proteins with enzymatic activity or solely as lectins. This could help bacteria to adapt and colonize the different environments encounter by ecological versatile microorganisms.

## Author Contributions

NV and PA: Substantial contributions to the conception and design of the work; acquisition, analysis, and interpretation of data for the work; drafting writing the work, final approval of the version to be published. DR: Acquisition, analysis, and interpretation of data for the work; drafting writing the work, final approval of the version to be published. EM and AL: Acquisition, analysis of data for the work; final approval of the version to be published. SM: Acquisition, analysis, and interpretation of data for the work; final approval of the version to be published. AZ: Substantial contributions to the conception and design of the work; analysis and interpretation of data for the work; drafting and writing the work, final approval of the version to be published.

## Conflict of Interest Statement

The authors declare that the research was conducted in the absence of any commercial or financial relationships that could be construed as a potential conflict of interest.

## Footnotes

1 http://www.bmrb.wisc.edu/metabolomics
